# Antitumor effects of L-BLP25 Antigen-Specific tumor immunotherapy in a novel human MUC1 transgenic lung cancer mouse model

**DOI:** 10.1186/1479-5876-11-64

**Published:** 2013-03-13

**Authors:** Gregory T Wurz, Audrey M Gutierrez, Brittany E Greenberg, Daniel P Vang, Stephen M Griffey, Chiao-Jung Kao, Michael Wolf, Michael W DeGregorio

**Affiliations:** 1 Department of Internal Medicine, Division of Hematology and Oncology, School of Medicine, University of California, Davis, 4501 X Street Suite 3016, Sacramento, CA, 95817, USA; 2 Comparative Pathology Laboratory, UC Davis School of Veterinary Medicine, University of California, Davis, CA, USA; 3 ImmunoOncology, Merck Serono Research, Merck KGaA, Germany

**Keywords:** L-BLP25, MUC1, Immunotherapy, Lung cancer, Cytokines

## Abstract

**Background:**

L-BLP25 antigen-specific cancer immunotherapeutic agent is currently in phase III clinical trials for non-small cell lung cancer. Using a novel human MUC1 transgenic (hMUC1.Tg) lung cancer mouse model, we evaluated effects of L-BLP25 combined with low-dose cyclophosphamide (CPA) pretreatment on Th1/Th2 cytokine production and antitumor activity.

**Methods:**

A chemically-induced lung tumor model was developed in hMUC1.Tg C57BL/6 mice by administering 10 weekly 0.75-mg/g doses of the chemical carcinogen urethane by intraperitoneal injection. Serum cytokines associated with Th1/Th2 polarization and inflammation were measured by multiplex cytokine assay during tumorigenesis. Antitumor activity of L-BLP25 (10 μg) with CPA (100 mg/kg) pretreatment was evaluated following either one or two eight-week cycles of treatment by preparing lung whole mounts and counting tumor foci, and assessing IFN-γ production by ELISpot assay.

**Results:**

During the carcinogenesis phase, no detectable Th1- or Th2-associated cytokine responses were observed, but levels of pro-inflammatory cytokines were increased with distinctive kinetics. A single cycle of L-BLP25 consisting of eight weekly doses was ineffective, whereas adding a second cycle given during tumor progression showed a significant reduction in the incidence of tumor foci. Administering two cycles of L-BLP25 induced Th1 cytokines IL-12, IL-2 and IFNγ at 24 h after the last dose, while Th2 and inflammatory cytokines were elevated to a lesser extent.

**Conclusions:**

Urethane-induced lung tumors in hMUC1.Tg mice can be used as a model to assess the efficacy of the MUC1 antigen-specific cancer immunotherapeutic agent L-BLP25. The results indicate that the antitumor response to L-BLP25 requires at least two cycles and pre-treatment with CPA. In addition, monitoring pro-inflammatory serum cytokines may be useful as a biomarker of L-BLP25 response. Taken together, the preclinical lung tumor model can be utilized for determining effective combinations of L-BLP25 with chemotherapy and/or other immunotherapies.

## Background

Lung cancer is the leading cause of cancer death in men and women, with an overall 5-year survival rate of approximately 10 to 15% [1]. The limited efficacy and the toxicity associated with chemotherapy for non-small cell lung cancer (NSCLC) has created a need for safer and more efficacious treatment options. With the identification of tumor-associated antibodies and antigens (TAA) in patients with lung cancer, immunotherapy has emerged as an attractive alternative [2,3].

Mucin 1 (MUC1) is one such TAA that is an epithelial glycoprotein overexpressed in NSCLC. T-cells specific for antigenic epitopes of MUC1 that bind to HLA class I molecules have been identified and isolated from the blood and bone marrow of cancer patients [4,5]. The immunodominant peptides from the variable number of tandem repeat region (VNTR) are recognized by the cytotoxic T-lymphocytes (CTL), making MUC1 an attractive target for therapeutic intervention [6]. A number of studies have shown that MUC1 may facilitate epithelial carcinogenesis [7-12]. High MUC1 expression in tumors has been correlated with increased invasiveness, migration, and angiogenesis in ovarian and lung cancers [7,9]. Depolarized expression of MUC1 has been related to poor prognosis in early stage NSCLC [8,10,11]. Recent findings have indicated that NSCLC cells are dependent on the MUC1-C terminal cytoplasmic domain for both activation of the phosphatidylinositol 3-kinase (PI3K)-Akt pathway and for survival [12].

A number of studies are focused on devising techniques to effectively present MUC1 as an immunogen to stimulate a strong and highly specific immune response against target cells overexpressing MUC1. L-BLP25 is one such innovative liposomal antigen-specific cancer immunotherapy currently under development that contains 25 amino acids from the immunogenic tandem**-**repeat region of MUC1 [13]. L-BLP25 is an active immunotherapeutic agent designed to induce a cellular immune response by targeting T-cell epitopes from the VNTR region of the MUC1 antigen associated with HLA class I molecules [14]. Although NSCLC is historically regarded as a non-immunogenic cancer [15], L-BLP25 in phase II clinical trials has shown survival advantages with a remarkably low toxicity profile [16,17]. In these trials a single, low, intravenous dose (300 mg/m^2^ to a maximum of 600 mg) of cyclophosphamide (CPA) is administered three days prior to immunotherapy. This procedure is thought to enhance delayed-type hypersensitivity humoral and cellular immune responses by reducing T-suppressor function. Although CPA lacks any significant activity in NSCLC [18], and the dose used in this setting is below that used in cytotoxic chemotherapy, it is unknown whether any of the observed antitumor effects following L-BLP25 therapy can be attributed to the immunomodulatory effects of CPA.

Recent gene profiling studies comparing signaling pathways in cancer among different species have detected marked similarities in mouse and human lung tumorigenesis [19,20]. Various approaches have been adopted to create a lung cancer mouse model, in which lung tumors may develop spontaneously or be induced with chemical carcinogens, transgenic oncogenes, viruses or radiation [21]. Unfortunately, there has been no well-established mouse model to study hMUC1-positive lung cancer. Rowse *et al.* produced a transgenic mouse model that expresses hMUC1 on an inbred C57BL/6 background [22]. However, C57BL/6 and DBA background mice are extremely resistant to lung carcinogenesis [23]. In spite of this limitation, previous studies in C57BL/6 mice have shown that 10 weekly injections of urethane at 1 mg/g caused a high incidence of lung adenomas [24]. In addition, Jiang *et al*. showed that diethylstilbestrol (DES) can promote the effects of urethane-induced mouse lung tumorigenesis in female Kunming mice [25].

The primary objectives of the current study were: (1) to develop a hMUC1-expressing lung tumor mouse model and utilize it to: (2) evaluate the effects of low-dose CPA on the L-BLP25-induced serum cytokine response; and (3) compare the antitumor activity of L-BLP25 with CPA pretreatment following one and two cycles of treatment.

## Methods

### Chemicals

Urethane and cyclophosphamide were purchased from Sigma-Aldrich® (St. Louis, MO). Diethylstilbestrol was purchased from Medisca (Plattsburgh, NY). L-BLP25 vaccine and the peptides used in ELISpot (BP25 and BP1-424) were provided by Merck Serono Research, Merck KGaA, Darmstadt, Germany.

### Animals

A total of 179 MUC1.Tg and 10 wild type C57BL/6 mice were supplied by our breeding colony maintained by the UC Davis Mouse Biology Program and housed at the UC Davis Center for Laboratory Animal Science vivarium. To establish the colony, heterozygous hMUC1 female transgenic founder mice were purchased from Mayo Clinic (Scottsdale, AZ). The mice were kept in cages (4 mice/cage) maintained at constant temperature and humidity with a 12-h light/12-h dark cycle. All mice received free access to water and Purina Laboratory Rodent Diet (LabDiet® 5001, PMI® Nutrition International, St. Louis, MO). All animal studies were conducted under a protocol approved by the University of California Davis Institutional Animal Care and Use Administrative Advisory Committee. UC Davis is an Association for Assessment and Accreditation of Laboratory Animal Care accredited institution. For all mouse studies, the week number refers to the age of the mice in each respective study.

### Genotype screening

For DNA extraction, toe clippings from two-week old mice were placed in a 96-well plate. Next, 40 μl of 50 mM NaOH was added to each well, and plates were heated to 94°C for 10 min. Extracted DNA was neutralized with 20 μl of 1 M Tris (pH 8) per well. Plates were sealed, vortexed briefly, centrifuged (6000 rpm × 2 min), and stored at −20°C until use. Polymerase chain reaction was used to identify MUC1.Tg mice using an ABI Prism 7900HT Sequence Detection System (AB Applied Biosystems, Carlsbad, CA). MTag forward and reverse primers were 5′-TTGGAGAATGTTTTTGTCTTGAA TG and 3′-CAGCACATCTCGGGTTGGT. The MTag TaqMan probe (ACATGCAATGGTTTGGAA) carrying a 6′ FAM reporter label and a 3′ MGBNFQ quencher group was used. For MUC1, forward and reverse primers were 5′-CACTCTTCCCCCAACCTTAAGTG and 3′-GGGTG GGTGGTGGTCATG. The MUC1 TaqMan probe (ACC AGTCCCTCCCTACG) carrying a 5′ VIC reporter label and a 3′ MGBNFQ was used*.* The amplification program consisted of one cycle of 2 min at 95°C and 40 cycles of 15 sec each at 60°C and 95°C.

### Immunohistochemistry (IHC)

The lungs, left kidney and spleen were harvested from each mouse at the time of sacrifice, which was performed by CO_2_ asphyxiation. The lungs were filled with 10% neutral buffered formalin prior to excision. All tissue samples were fixed in 10% buffered formalin overnight, followed by 70% ethanol until processed. Tissues were then paraffin embedded and step-sectioned at 4 μm for immunohistochemical analysis. Immunohistochemistry was performed using a MUC1 antibody (CD227, 550486; 1:400; BD Pharmingen) which recognizes the tandem-repeat region. The Animal Research Kit peroxidase (ARK; K3954; Dako) was used to minimize reactivity of secondary mouse antibody with endogenous immunoglobulin present in the tissue. Lung whole mounts were prepared using standard protocols.

### Multiplex cytokine assays

The Mouse Cytokine 20-plex Panel (Invitrogen; cat. #LMC0006, Carlsbad, CA) was used to analyze the levels (pg/mL) of Th1/Th2 and inflammatory cytokines in all serum samples except for those from the studies described under “Different Schedules of L-BLP25”, for which a 25-plex Milliplex MAP Mouse Cytokine/Chemokine Magnetic Bead Panel (Millipore, Billerica, MA) was used. The 20-plex panel consisted of interleukin (IL) -1α, IL-1β, IL-2, IL-4, IL-5, IL-6, IL-10, IL-12, IL-13, IL-17, interferon gamma (IFN-γ), interferon gamma-induced protein 10 (IP-10), monokine induced by IFN-γ (MIG), keratinocyte derived cytokine (KC), monocyte chemotactant protein-1 (MCP-1), macrophage inflammatory protein-1 alpha (MIP-1α), granulocyte/macrophage colony stimulating factor (GM-CSF), vascular endothelial growth factor (VEGF), tumor necrosis factor alpha (TNF-α), and basic fibroblast growth factor (FGF-basic). In addition to the analytes listed for the 20-plex panel, with the exception of VEGF, MIG, and FGF-basic, the 25-plex panel included granulocyte colony-stimulating factor (G-CSF), IL-7, IL-9, IL-12 (p40/p70), IL-15, MIP-1β, MIP-2, and regulated on activation, normal T-cell expressed and secreted (RANTES). The assays were performed according to their respective manufacturer’s instructions. The concentration of each cytokine was calculated relative to respective standard curves. For 20-plex analyses, cytokine concentrations were acquired on a BioPlex System using BioPlex software version 5.0 (BioRad, Hercules, CA, USA). The 25-plex analysis was performed on a Luminex 100/200 system running xPonent software version 3.1 (Luminex Corporation, Austin, TX).

#### In-house control

To establish the in-house control for this assay, wildtype C57BL/6 mice were challenged with lipopolysaccharide (LPS). Mice were injected intraperitoneally (i.p.) with 200 μg LPS from *Escherichia coli* serotype O111:B4 (Sigma-Aldrich) dissolved in 1X PBS. At 4–5 h post-injection, mice were euthanized by CO_2_ asphyxiation. Whole blood was collected by cardiac puncture and placed in a clotting tube for isolation of serum by centrifugation. The serum was flash frozen in liquid nitrogen and stored at −80°C until analysis by multiplex assays on Luminex system.

### Lung cancer model development in MUC1.Tg mice

For this study, 38 male MUC1.Tg mice (5 weeks of age) were divided into five groups: Control (vehicle only) (n = 8); Urethane (n = 10); Urethane + DES 7 mg/kg (n = 10); and Urethane + DES 14 mg/kg (n = 10). A second urethane group composed of 10 wild type males was included to compare human MUC1 expression with the transgenic mice. Urethane (0.75 mg/g) or sterile water (control) was administered by i.p. injection weekly for 10 weeks using a 25-gauge needle. All injection volumes were 100 μl. Starting 24 h after urethane dose 4, DES (7 mg/kg or 14 mg/kg) or corn oil (control) was administered weekly for a total of 8 weeks by subcutaneous (s.c.) injection in a volume of 100 μl using a 25-gauge needle. Mice were monitored until termination of the study in Week 38. The lungs, spleens, and left kidneys were collected and analyzed from a total of 46 mice. The presence of hMUC1-expressing lung adenoma was confirmed by blinded IHC evaluations performed by a pathologist.

### Cytokine response in MUC1.Tg mice

We studied the polarization of cytokine response in the hMUC1.Tg model during the induction and progression stages of lung tumor development. A total of six male hMUC1.Tg mice were bled 24 h after the 5^th^, 8^th^, and 10^th^ doses of urethane (Weeks 10, 12, and 15, respectively) and thereafter at approximately four-week intervals (Weeks 20–40). Urethane administration was performed starting at approximately 4 weeks of age, as described under Lung Cancer Model Development in MUC1.Tg mice. Whole blood was collected from mice via submandibular bleeds, pooled and allowed to clot for 30 min, and then serum was isolated by centrifugation for 10 min at 3500 × g. Serum was flash frozen in liquid nitrogen and stored at −80°C until analysis by Luminex assay.

### Effect of single dose of Cyclophosphamide on Th1/Th2 cytokine response

To determine the effect of low-dose CPA on the L-BLP25-induced immune response, and whether low-dose CPA induces a Th2/Th1 cytokine shift in this model, a total of 103 MUC1.Tg mixed sex C57BL/6 mice were divided into five treatment groups: Control (n = 20), CPA 100 mg/kg (n = 21), CPA 300 mg/kg (n = 20), L-BLP25 alone (n = 21) and CPA 100 mg/kg + L-BLP25 (n = 21). Each group contained 13 male and 8 female mice, except for the control and CPA 300 mg/kg groups, which each contained seven females. All mice underwent 10 weeks of urethane dosing at 0.75 mg/g weekly starting at 4 weeks of age. At Week 14, four days after urethane dosing was completed, mice were administered a single dose of CPA at either 100 or 300 mg/kg by i.p. injection according to treatment group assignment. Whole blood was collected from mice in each treatment group via submandibular bleeds 24 h following CPA administration. In our L-BLP25 mouse studies we have been using a CPA dose of 100 mg/kg, which is equivalent to the 300-mg/m^2^ dose used in humans [26]. Three days after CPA administration, mice were given s.c. injections of 10 μg L-BLP25 at rotating sites according to treatment group using a 25-gauge needle once each week for eight weeks, with the 8th dose being administered in Week 21. The lyophilized L-BLP25 was reconstituted in sterile 0.9% saline to a concentration of 100 μg/ml and delivered in a volume of 100 μl. Twenty-four hours after the 4^th^ and 8^th^ doses of L-BLP25, whole blood was collected from mice in each treatment group via submandibular bleeds. Blood was pooled within a treatment group and serum was isolated by centrifugation. The serum was flash frozen in liquid nitrogen and stored at −80°C until analysis by Luminex assay. In order to assess the antitumor effects of CPA when combined with L-BLP25, this study was continued through Week 41 as described below in the two-cycle dosing study.

### Different schedules of L-BLP25

Two studies were conducted in order to determine the effects of L-BLP25 on the development of lung tumors in this mouse model following either one or two cycles of treatment. In the first study, 32 MUC1.Tg male C57BL/6 mice were divided into four treatment groups: Untreated; CPA 100 mg/kg; L-BLP25 alone; and L-BLP25 + CPA 100 mg/kg (n = 8, all groups). All mice received 10 weekly i.p. injections of 0.75 mg/g urethane starting at four weeks of age as described under model development. Cyclophosphamide was then prepared and administered as described above, 24 h following the final urethane dose (Week 13). A single eight-dose cycle of weekly L-BLP25 was begun three days following CPA administration (Week 14). L-BLP25 was prepared as described above and administered weekly for a total of eight 10-μg doses by s.c. injection at rotating sites using a 25-gauge needle (injection volume 100 μl). Mice were then followed for 20 additional weeks. At the conclusion of the study in Week 41, all mice were euthanized by CO_2_ asphyxiation. Whole blood was then collected by cardiac puncture, and lung whole mounts were prepared to compare average lung tumor numbers between groups. Serum was isolated from whole blood and stored as described above.

In the two-cycle L-BLP25 study, which was a continuation of the low-dose CPA study described above, a total of 65 male and female MUC1.Tg C57BL/6 mice in four treatment groups were utilized. To reiterate, the treatment groups were Untreated (n = 18), CPA 100 mg/kg (n = 17), L-BLP25 alone (n = 14), and L-BLP25 + CPA 100 mg/kg (n = 16). The CPA 300 mg/kg group was excluded. This study was designed as described for the single-cycle study, except that 11 weeks following the end of the first eight-dose cycle of L-BLP25 (Week 32), these mice began a second cycle of treatment that continued weekly until the conclusion of the study in Week 41. The second cycle consisted of a total of nine 10-μg doses of L-BLP25, which was prepared and administered as described above. Three days prior to beginning the second cycle of L-BLP25, another 100-mg/kg dose of CPA was administered. At the end of the study, mice were euthanized by CO_2_ asphyxiation, and whole blood was collected by cardiac puncture. Serum was isolated and stored as described above for Luminex system analysis. Following blood collection, lung whole mounts were prepared to compare total average tumor numbers between treatment groups. IFN-γ/IL-4 ELISpot analysis was performed as detailed below on four mice from each treatment group to assess immune response.

### ELISpot

To assess the immune response to L-BLP25 following one and two cycles of treatment, splenocytes from L-BLP25-treated and untreated mice were examined for Th1/Th2 polarization by analyzing two key cytokines: IFN-γ for Th1 and IL-4 for Th2 polarization. Dual color ELISpot mouse IFN-γ/IL-4 (R&D Systems, Minneapolis, MN) kits were used for this assay. Spleens were removed aseptically, processed through 100-μm nylon tissue sieves (Becton Dickinson, Franklin Lakes, NJ) into sterile PBS, and the cell suspensions were layered over lymphocyte separation medium (Lonza, Walkersville, MD). Lymphocytes were isolated by centrifuging at 600 × g for 15 min, washed in PBS, and then resuspended in improved minimum essential medium (Invitrogen) containing 10% fetal bovine serum (HyClone, Logan, UT), 50 μg/ml streptomycin and 50 U/ml penicillin (Invitrogen) prior to counting and viability assessment using an Auto T4 Cellometer™ (Nexcelom Bioscience, Lawrence, MA). BP25 peptide and scrambled peptide (BP1-424) were prepared at a final concentration of 5 μg/ml in culture medium. Lymphocytes (1.0 × 10^6^/well) were incubated with either no peptide (medium only), BP25, or scrambled peptide in triplicate at 37°C overnight. ELISpot plates were developed according to the manufacturer’s instructions.

### Statistical methods

Differences in tumor incidence between experimental groups in the model development study were evaluated for statistical significance using Fisher’s exact test (two-tailed). In the one- and two-cycle L-BLP25 studies, a one-way ANOVA was used for comparisons between average number of lung tumors between the various treatment groups. Bonferroni’s adjustment for multiple comparisons was used to lessen the likelihood of a false positive result. GraphPad Prism® 5 software was used for all statistical analyses. A p-value of ≤ 0.05 was considered significant for all analyses.

## Results

### Urethane induces hMUC1-expressing lung adenomas

MUC1.Tg mice treated with urethane alone developed lung adenomas with a tumor incidence rate of 100%, while urethane in combination with DES resulted in tumor incidence rates of 50% and 67% at the 7- and 14-mg/kg DES dose levels, respectively. Lung tumor incidence following treatment with urethane alone was significantly greater than urethane combined with 7 mg/kg DES (*p* = 0.0302), but not urethane plus 14 mg/kg DES. These results indicate that the addition of DES treatment did not increase tumor incidence, in contrast to Jiang *et al*. [25]. Furthermore, it is evident that urethane alone at a dose of 0.75 mg/g injected weekly for 10 weeks was sufficient to induce lung adenomas in MUC1.Tg C57BL/6 mice.

### Histopathological evaluation

**Figure 1:**
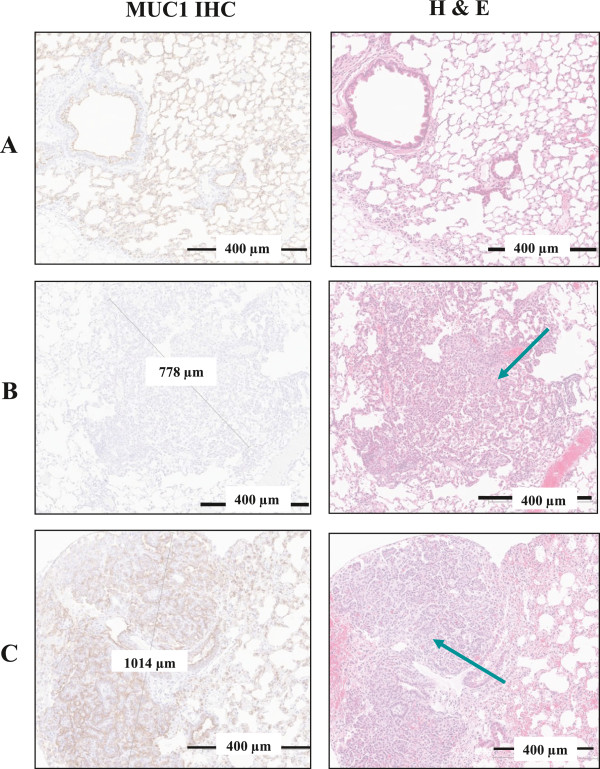
**Comparison of hMUC1 expression in lung tissues and tumors from MUC1.Tg vs. wild type mice at week 38.** (**A**) Lung tissue from a MUC1.Tg control mouse exhibiting hMUC1 positivity and normal H&E staining. (**B**) Lung tissue from a urethane-treated wild type mouse exhibiting hMUC1-negative adenoma; the H & E staining shows a representative tumor region (arrow). (**C**) Lung tissue from a urethane-treated MUC1.Tg mouse exhibiting hMUC1-positive adenoma; the H&E staining shows a representative tumor region (arrow). Magnification = 60X (all panels). A number of samples showed tumors with multiple foci.

As shown in Figure 1, histopathological evaluation of urethane-treated mouse lungs revealed adenoma with high expression of hMUC1. Urethane exposure consistently resulted in pulmonary neoplasms of varying size and with some variation in phenotype ranging from papillary to trabecular and solid. Neoplastic foci were seen in both central and peripheral areas of the lung parenchyma, but consistently arose in alveolar spaces, and not from larger bronchi. By comparison, these foci appear most similar to human bronchioloalveolar carcinomas, and in the earliest stages (smallest foci) are similar to human atypical adenomatous foci. Arbitrary size criteria have been used traditionally to divide rodent pulmonary neoplasms into adenoma or carcinoma, but more recently, recommendations from the NCI Mouse Models of Human Cancers Consortium consensus meeting on mouse models of lung cancer has recommended against using the term “bronchioloalveolar” when referring to mouse lung lesions in favor of the more simplified terms of adenoma or adenocarcinoma [27]. All sections were reviewed blinded to cohort conditions. All spleens from the MUC1-expressing mice were negative and all kidneys positive for hMUC1 expression.

### Time course of cytokine response during tumor development

**Figure 2:**
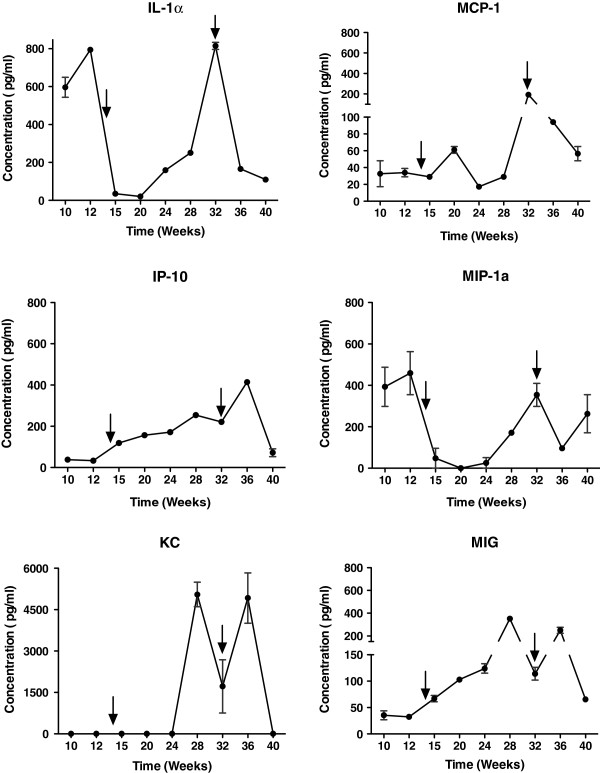
**Pro-inflammatory cytokines at different stages of tumor progression.** Serial serum specimens were collected via submandibular bleeds following urethane doses 5, 8, and 10 and then every four weeks thereafter until study termination. Blood was pooled (n = 6), and the serum was isolated and analyzed for the presence of 20 cytokines. Concentrations represent the mean of pooled samples and bars represent the range. Arrows indicate the points at which L-BLP25 cycles one and two were begun in the L-BLP25 cycle-dependent studies.

We studied the polarization of cytokine response in MUC1.Tg mice at different phases of tumor development. Mice were bled approximately 24 h after the 5^th^, 8^th^ and 10^th^ doses of urethane, and thereafter at approximately 4-week intervals. Cytokines were grouped into Th1 type (IL-2, IL-12, GMCSF, IFN-γ, TNF-α, IL-17), Th2 (IL-4, IL-5, IL-6, IL-10, IL-13), and inflammatory/chemokine type (IL-1α, IL-1β, IP-10, MIG, KC, MCP-1, MIP-1α). The Th1 cytokines IL-2, IL-12, IL-17, TNF-α, GMCSF and IFN-γ, and Th2 cytokines IL-4, IL-5, IL-6, IL-10 and IL-13 demonstrated no consistent pattern (data not shown), suggesting that urethane has minimal effects on Th1/Th2 cytokine polarization. Figure 2 shows the levels of pro-inflammatory cytokines at different stages of model development. Potent inflammatory cytokines IL-1α and MIP-1α were elevated during Week 10, which may be result of an inflammatory response to urethane exposure. Levels of these cytokines and other pro-inflammatory cytokines such as IP-10, MIG, KC, MCP-1, and MIP-1α were elevated post Week 20 (see Figure 2), which may be a result of an inflammatory response associated with tumor progression.

### Single Low-dose Cyclophosphamide potentiates L-BLP25-induced Th1 cytokine response

In order to evaluate the effect of low-dose CPA on the L-BLP25-induced Th1 cytokine response, we utilized a multiplex assay to analyze serum collected 24 h after the 4^th^ and 8^th^ weekly doses of L-BLP25. Cytokines analyzed were grouped as follows: Th1 type (IL-2, IL-12, IFN-γ), Th2 (IL-4, IL-5, IL-6, IL-13), and inflammatory/chemokine type (IP-10, MIG, KC, MCP-1, MIP-1α). Using this multiplex assay we found noticeable differences in serum cytokine levels of L-BLP25-treated mice compared to mice given the combination of CPA 100 mg/kg and L-BLP25.

**Figure 3:**
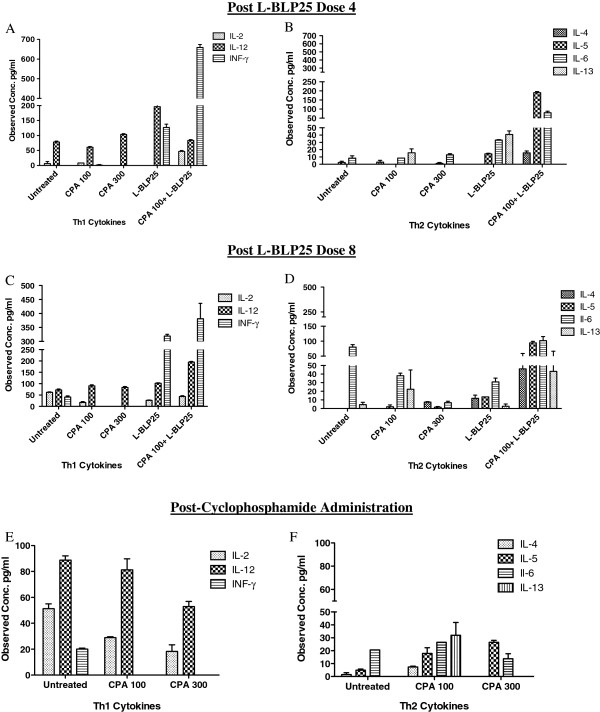
**Effects of CPA alone and in combination with L-BLP25 on serum Th1/Th2 cytokines.** Serial whole blood specimens were collected via submandibular bleeds 24 hours after the 4^th^ (**A**, **B**) and 8^th^ (**C**, **D**) doses of L-BLP25, and 24 hours following the 100- or 300-mg/kg CPA administrations (**E**, **F**). Blood was pooled within each treatment group (n = 3-6 depending on blood volume obtained), and the serum was isolated and analyzed for the presence of 20 cytokines. Serum concentrations represent the mean and bars represent the range. The serum concentrations of IFN-γ were noticeably higher in the mice treated with CPA + L-BLP25 compared to those treated with L-BLP25 alone.

For Th1 cytokines, the levels of IL-2 and IFN-γ showed an elevated trend in the CPA + L-BLP25 treated mice in comparison to L-BLP25 alone 24 h following the 4^th^ and 8^th^ doses (Figure 3A and 3C). For IL-12, the levels were elevated in the L-BLP25 alone treatment group compared to the CPA 100 mg/kg + L-BLP25 group 24 h post-dose 4 (Figure 3A), whereas the post-dose 8 levels were elevated in the CPA 100 mg/kg + L-BLP25 group compared to the L-BLP25 alone group (Figure 3C). This discrepancy could be a result of samples being pooled together within a treatment group. Although the IL-12 levels were inconclusive, the post-L-BLP25 dose 4 levels of two other main Th1 cytokines (IL-2 & IFN-γ) were elevated in the CPA 100 mg/kg + L-BLP25 group in comparison to the L-BLP25 alone group, and a similar trend was observed following dose 8. While some of the Th2 cytokines also showed an elevated trend in the CPA + L-BLP25 treated mice in comparison to mice treated with L-BLP25 alone following the 4th and 8th dose (Figures 3B and 3D), on balance the predominant immune response was Th1 polarized, as evidenced by the noticeably elevated IFN-γ levels.

**Figure 4:**
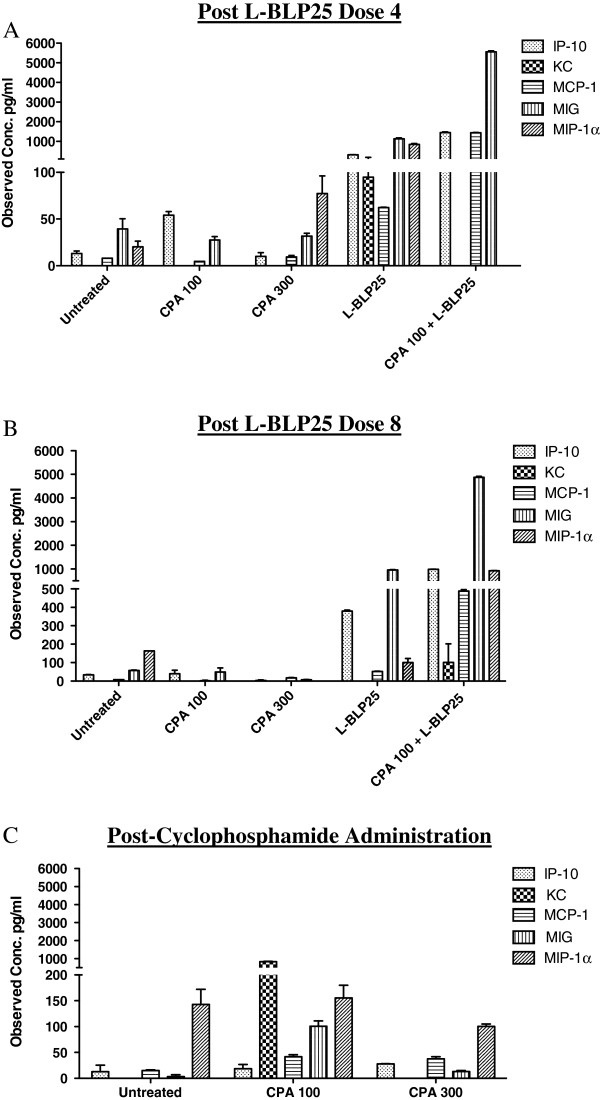
**Effects of CPA alone and in combination with L-BLP25 on serum pro-inflammatory cytokines.** Serial serum specimens were analyzed for 20 cytokines 24 hours after the 4^th^ (**A**) and 8^th^ (**B**) doses of L-BLP25, and 24 hours after the CPA 100- and 300-mg/kg doses (**C**). Blood was pooled within each treatment group (n = 3-6, depending on blood volume obtained). For each treatment group, serum concentrations represent the mean and bars represent the range. Serum concentrations of IP-10, MIG, MIP-1α and MCP-1 were elevated in the CPA + L-BLP25 group compared to vaccine alone.

Levels of inflammatory cytokines (IP-10, MIG, KC, MCP-1, MIP-1α) trended towards being elevated in the CPA 100 mg/kg + L-BLP25 group in comparison to the L-BLP25 alone group (Figures 4A and 4B). This could be due to elevated levels of IFN-γ seen in the CPA 100 mg/kg + L-BLP25 group. Inflammatory chemokines like IP-10 and MIG are known chemoattractants for T-lymphocytes [28].

To determine the effect of low versus high, cytotoxic-dose CPA on serum Th1/Th2 cytokine polarization as well as on inflammatory cytokines, we analyzed serum samples collected 24 h following administration of the 100 mg/kg and 300 mg/kg doses of CPA. No evidence supporting a Th2 to Th1 shift in cytokine response following low-dose or high-dose CPA administration was observed (Figures 3E and 3F). For inflammatory cytokines, the levels of KC and MIG were elevated in the 100 mg/kg group compared to the 300 mg/kg group (Figure 4C).

### Cycle-dependent effects of L-BLP25 on tumor incidence

**Figure 5:**
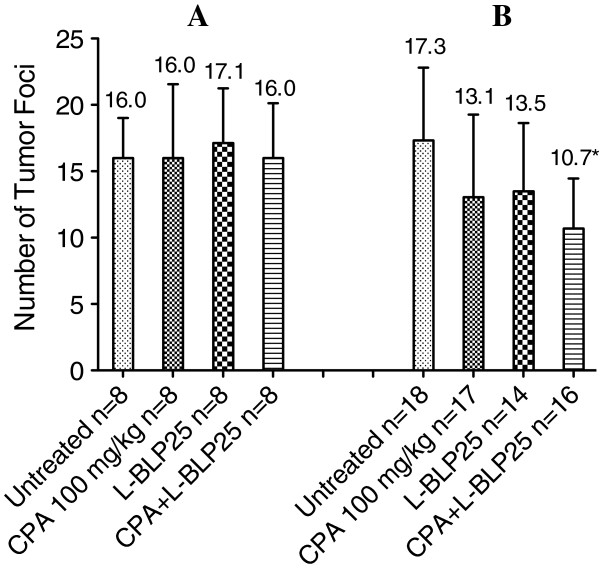
**Cycle-dependent antitumor activity of L-BLP25.** In the single-cycle study (**A**), male MUC1.Tg mice were given one cycle of eight weekly doses of L-BLP25 starting immediately following urethane induction and then followed for 20 additional weeks. In the two-cycle study (**B**), MUC1.Tg mice were given an additional cycle of L-BLP25 starting 11 weeks after the end of the first cycle and continuing weekly until the conclusion of the study in Week 41. The only statistically significant difference in number of tumor foci (p < 0.01) was observed between the untreated and CPA + L-BLP25 groups following two cycles of L-BLP25 (**B**). In both studies, CPA was administered three days prior to the start of each cycle of L-BLP25. Data are shown as average number of tumor foci + standard deviation.

No significant differences in the number of tumor foci were observed following eight weekly treatments with L-BLP25, either alone or with CPA pretreatment (Figure 5). In the two-cycle dosing study, however, a significant reduction in the number of tumor foci was observed in the L-BLP25 + CPA 100 mg/kg treatment group compared to the untreated group (p < 0.01). Interestingly, both the L-BLP25 alone and CPA 100 mg/kg groups in the two-cycle study showed a trend toward a reduced number of tumor foci compared to the untreated group, although statistical significance was not achieved (Figure 5). This result suggests that adding a second cycle of CPA and/or L-BLP25 administration may be important with respect to lung tumor development. As shown in Figure 2, serum levels of inflammatory cytokines were flat in Week 14 when L-BLP25 treatment was begun in the single-cycle study; however, when the second cycle of L-BLP25 treatment was begun in Week 32 in the two-cycle study, serum concentrations of these cytokines were much higher, which may be associated with lung tumor development. This observation may explain in part the antitumor activity of L-BLP25 treatment in the two-cycle study.

**Figure 6:**
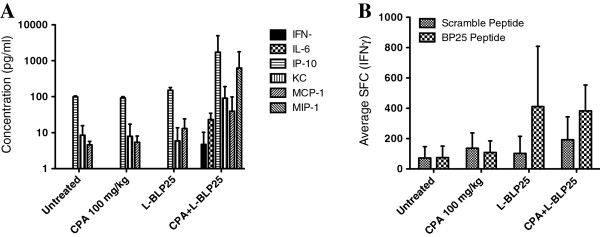
**IFN-γ immune response and Th1, Th2, and average inflammatory serum cytokine concentrations in the L-BLP25 two-cycle study.** Luminex (**A**) and ELISpot (**B**) analyses were performed on four mice from each treatment group at the conclusion of the study (Week 41). Serum samples from individual mice were analyzed for the presence of 25 cytokines. For all analyses, n = 4. SFC = spot forming colonies. Error bars represent positive standard deviation.

Figure 6 shows the serum concentrations of Th1, Th2, and inflammatory cytokines (Figure 6A), as well as the corresponding IFN-γ immune response in the 16 mice used in the ELISpot analysis for the two-cycle study (Figure 6B). Although L-BLP25 alone and with CPA pretreatment produced a specific IFN-γ immune response, only L-BLP25 with CPA pretreatment showed detectable serum levels of IFN-γ (Figure 6A). Taken together with the tumor foci data, these results suggests that L-BLP25 with CPA pretreatment is more effective than L-BLP25 alone, and that the effects of L-BLP25 on lung tumor control require at least two cycles of treatment. Ongoing studies are designed to correlate inflammatory serum cytokines and tumor foci to overall survival following multiple cycles of L-BLP25.

## Discussion

It is well known that the biologic effects of low-dose CPA differ from higher, cytotoxic doses. The immunomodulating capacity of low-dose CPA has been shown to reduce both the number of T-regulatory cells (T-regs) [29-33] and their immunosuppressive functionality in mice [32] and humans [30]. Based on these observations, CPA at a low dose of 300 mg/m^2^ is being used in all human L-BLP25 clinical trials with the rationale of potentiating the immune response to L-BLP25. Previous studies have shown that low-dose CPA augments delayed-type hypersensitivity (DTH) responses to antigen by selectively suppressing CD4 + CD25+ T-reg cells while having relatively little effect on CD8+ cytotoxic T-cells or the CD4+/CD8+ cell ratio [28-36]. T-regulatory cells are known to suppress the immune response against tumors [29,37,38]. Higher, cytotoxic doses of CPA impact all of the T-lymphocyte populations, resulting not only in reduced Treg counts but also reduced CD4+, CD8+, and total splenocyte counts [29,33]. Thus, a cytotoxic dose of CPA can impair the immune response to tumor-associated antigens [29]. These observations are consistent with Motoyoshi *et al.* who showed that low-dose CPA produced an antitumor immune response by selectively depleting Tregs in immunocompetent mice, while the effects of high-dose CPA were attributable solely to its cytotoxic effects [33]. In the present study, we showed that pretreatment with low-dose CPA (100 mg/kg) potentiated the L-BLP25-induced Th1 cytokine response, as demonstrated by elevated IFN-γ and IL-2 levels. These results are in accordance with previous findings by Machiels *et al.* who demonstrated that CPA, when given in a defined sequence with a GM-CSF-secreting, neu-expressing whole-cell vaccine, enhanced the efficacy of the vaccine and appeared to amplify the Th1 neu-specific T-cell response [39]. The combination of CPA and L-BLP25 has already been employed in clinical trials in non-small cell lung cancer.

The malignant potential and progression of the urethane-induced tumors characterized in this study cannot be easily predicted. In many cases, urethane-induced lung tumors in mouse models are adenomas and do not progress to adenocarcinomas with metastasis. However, this model may be advantageous for preclinical testing, given that cancer vaccines seem to be more effective in patients with low tumor burden or indolent disease [40]. Although lung cancer is notoriously difficult to treat, even in its early stages, the fact that a modest yet significant reduction in the number of tumor foci following two cycles of L-BLP25 treatment was encouraging. In view of this observation, our model may prove more clinically relevant in determining the most effective combinations of L-BLP25 and chemotherapeutic agents or other immunotherapies. For example, the timing of L-BLP25/CPA combination therapy may be critical with respect to antitumor activity. The kinetics of serum pro-inflammatory cytokines may be associated with tumor development and progression, which in turn may be useful in tailoring the timing of immunotherapy/chemotherapy treatment regimens. In the two-cycle L-BLP25 study, reductions in the numbers of tumor foci were associated with treatments during a period when serum pro-inflammatory cytokines were increasing.

## Conclusions

Although it was recently announced that the phase III trial of L-BLP25 in patients with unresectable, locally advanced stage IIIA or IIIB non-small cell lung cancer did not meet its primary endpoint of increasing overall survival, important scientific insights to the potential for immunotherapies were noted. In the present preclinical study, we showed a rather modest antitumor response, which is consistent with the primary endpoint result of the phase III trial. However, only the publication of the detailed results of the phase III trial will allow further comparisons and conclusions between patients or subgroups of patients and this preclinical model. Translating preclinical research also presents theoretical problems, as we recently summarized [41]. The lung cancer model described here offers a platform for developing effective immunotherapy combinations, as well as combinations of immunotherapy with targeted therapy. Future studies examining inflammatory cytokines as biomarkers and surrogate measures of tumor development and tailoring immunotherapy treatment to a cytokine response are warranted.

## Abbreviations

CPA: Cyclophosphamide; CTL: Cytotoxic T-lymphocyte; DES: Diethylstilbestrol; DTH: Delayed-type hypersensitivity; FGF-basic: Basic fibroblast growth factor; GM-CSF: Granulocyte/macrophage colony stimulating factor; HLA: Human lymphocyte antigen; hMUC1.Tg: Human MUC1 transgenic; IFN-γ: Interferon gamma; IHC: Immunohistochemistry; IL: Interleukin; i.p.: Intraperitoneal; IP-10: Interferon gamma-induced protein 10; KC: Keratinocyte derived cytokine; LPS: Lipopolysaccharide; MCP-1: Monocyte chemotactant protein-1; MIG: Monokine induced by IFN-γ; MIP-1α: Macrophage inflammatory protein-1; MUC1: Mucin 1; NCI MMHCC: National Cancer Institute Mouse Models of Human Cancers Consortium; NSCLC: Non-small cell lung cancer; PBS: Phosphate buffered saline; RANTES: Regulated on activation, normal T-cell expressed and secreted; s.c.: Subcutaneous; SFC: Spot-forming colony; TAA: Tumor-associated antigen; Th1/Th2: T-helper 1/T-helper 2; TNF-α: Tumor necrosis factor alpha; T-reg: T-regulatory cell; VEGF: Vascular endothelial growth factor; VNTR: Variable number of tandem repeats.

## Competing interests

GTW, AMG, BEG, DPV, CJK, and SMG declare no competing interests. MWD is the Principal Investigator of a grant received from Merck KGaA, and MW is an employee of Merck KGaA.

## Authors’ contributions

GTW prepared the manuscript and participated in the conduct and design of the various studies. AMG, BEG and DPV participated in the conduct of the various studies and performed the multiplex, ELISpot, statistical analyses and editing the manuscript. SMG performed the histopathological assessments and editing the manuscript. CJK participated in the study design, interpretation of results and editing the manuscript. MW participated in the design of the various studies and editing the manuscript. MWD conceived and designed the various studies and editied the manuscript. All of the authors read and approved the final manuscript.

## Authors’ information

GTW has a Ph.D. in pharmacology/toxicology and is a research scientist at UC Davis. AMG has a Ph.D. in immunology and is currently a post-doctoral scholar at UC Davis. BEG has a B.S. in animal science and is currently a junior specialist at UC Davis. DPV is an expert in cytokine multiplex analysis, has a B.S. and is currently a research scientist at UC Davis. SMG is a pathologist at UC Davis and has a DVM in addition to a Ph.D. in pathology. CJK has a Ph.D. in molecular biology and is a visiting Scholor at UC Davis. MW has a Ph.D. in immunology and is presently head of applied immunology at Merck Serono Research, division of ImmunoOncology, Merck KGaA. MWD has a Pharm.D. and is a professor of medicine at UC Davis.
